# Prostatic Artery Embolization Versus Transurethral Resection of the Prostate: A Post Hoc Cost Analysis of a Randomized Controlled Clinical Trial

**DOI:** 10.1007/s00270-021-02920-3

**Published:** 2021-07-20

**Authors:** Ferran Capdevila, Iñigo Insausti, Arkaitz Galbete, Eduardo Sanchez-Iriso, Manuel Montesino

**Affiliations:** 1 Complejo Hospitalario de Navarra, Calle Irunlarrea 3, 31008 Pamplona, Spain; 2 Universidad Pública de Navarra (UPNA), Campus de Arrosadia, s/n, 31006 Pamplona, Spain; 3 Instituto de Investigación Sanitaria de Navarra (IdiSNA), Calle Irunlarrea 3, 31008 Pamplona, Spain; 4 Clínica Universidad de Navarra, Av. de Pío XII, 36, 31008 Pamplona, Spain; 5 Navarrabiomed, Calle Irunlarrea 3, 31008 Pamplona, Spain; 6 Red de Investigación en Servicios de Salud en Enfermedades Crónicas (REDISSEC), Madrid, Spain

**Keywords:** Prostatic artery embolization (PAE), Transurethral resection of the prostate (TURP), Costs, Benign prostatic hyperplasia

## Abstract

**Purpose:**

To perform a post hoc analysis of patient-incurred costs in a randomized controlled clinical trial comparing prostatic artery embolization (PAE) and transurethral resection of the prostate (TURP).

**Materials and Methods:**

Patients older than 60 years with indication of TURP were randomized to PAE or TURP procedure. After intervention and hospital discharge, patients were follow-up during 12 months The associated patient costs were categorized according to the study period: pre-intervention, intervention, hospitalization, and follow-up. Several items for both groups were analyzed within each study period.

**Results:**

The mean total costs per patient were lower for PAE (€ 3,192.87) than for TURP (€ 3,974.57), with this difference of € 781.70 being significant *(p* = 0.026). For most evaluated items, the mean costs were significantly higher for TURP. No significant differences were observed in the mean costs of PAE (€ 1,468.00) and TURP (€ 1,684.25) procedures (*p* = 0.061). However, the histopathology analysis, recovery room stay, and intraoperative laboratory analysis increased the interventional costs for TURP (€ 1,999.70) compared with PAE (€ 1,468.00) (*p* < 0.001). No cost differences were observed between PAE (€ 725.26) and TURP (€ 556.22) during the 12 months of follow-up (*p* = 0.605). None of patients required a repeat intervention during the study period.

**Conclusions:**

Considering the short-term follow-up, PAE was associated with significantly lower costs compared with TURP. Future investigations in the context of routine clinical practice should be aimed at comparing the long-term effectiveness of both procedures and determining their cost-effectiveness.

Level of evidence: Level 1 (a-c)

## Introduction

Benign prostatic hyperplasia (BPH) is characterized by the nonmalignant overgrowth of prostatic tissue surrounding the urethra, and is a common cause of lower urinary tract symptoms (LUTS) in men. This condition is present in approximately 8% of men aged between 31 and 40 years. This prevalence increases significantly with age, to approximately 90% by the eighth decade of life [1]. Surgical management is indicated when pharmacological treatment is not tolerated or the disease is refractory to it. Transurethral resection of the prostate (TURP) is the gold-standard surgical treatment for patients with BPH with prostate sizes of 30–80 mL and bothersome moderate-severe LUTS secondary to benign prostatic obstruction [2].

Prostatic artery embolization (PAE) has emerged as a minimally invasive therapy for symptomatic patients due to BPH and as an alternative to TURP, with similar clinical outcomes, fewer complications, and shorter recovery time [3–9]. The National Institute for Health and Care Excellence (NICE) supports the use of PAE for LUTS secondary to BPH, provided that standard arrangements are in place for clinical management, consent and audit [10].

Considering the substantial differences between the technical aspects of PAE and TURP, there may also be differences in their costs, which is why we believe that a cost analysis comparing each procedure seems to be of particular interest. To date, two cost analysis are available: Bagla et al. [11] and Müllhaupt G et al. [12]. Both studies found that PAE was associated with lower interventional costs. However, the costs of follow-up, complications or the medication involved were not considered. The aim of this study was to analyze patient-incurred costs in a randomized controlled clinical trial comparing PAE and TURP performed in a hospital of the Spanish National Health System and with a twelve months follow-up.

## Materials and Methods

**Fig. 1:**
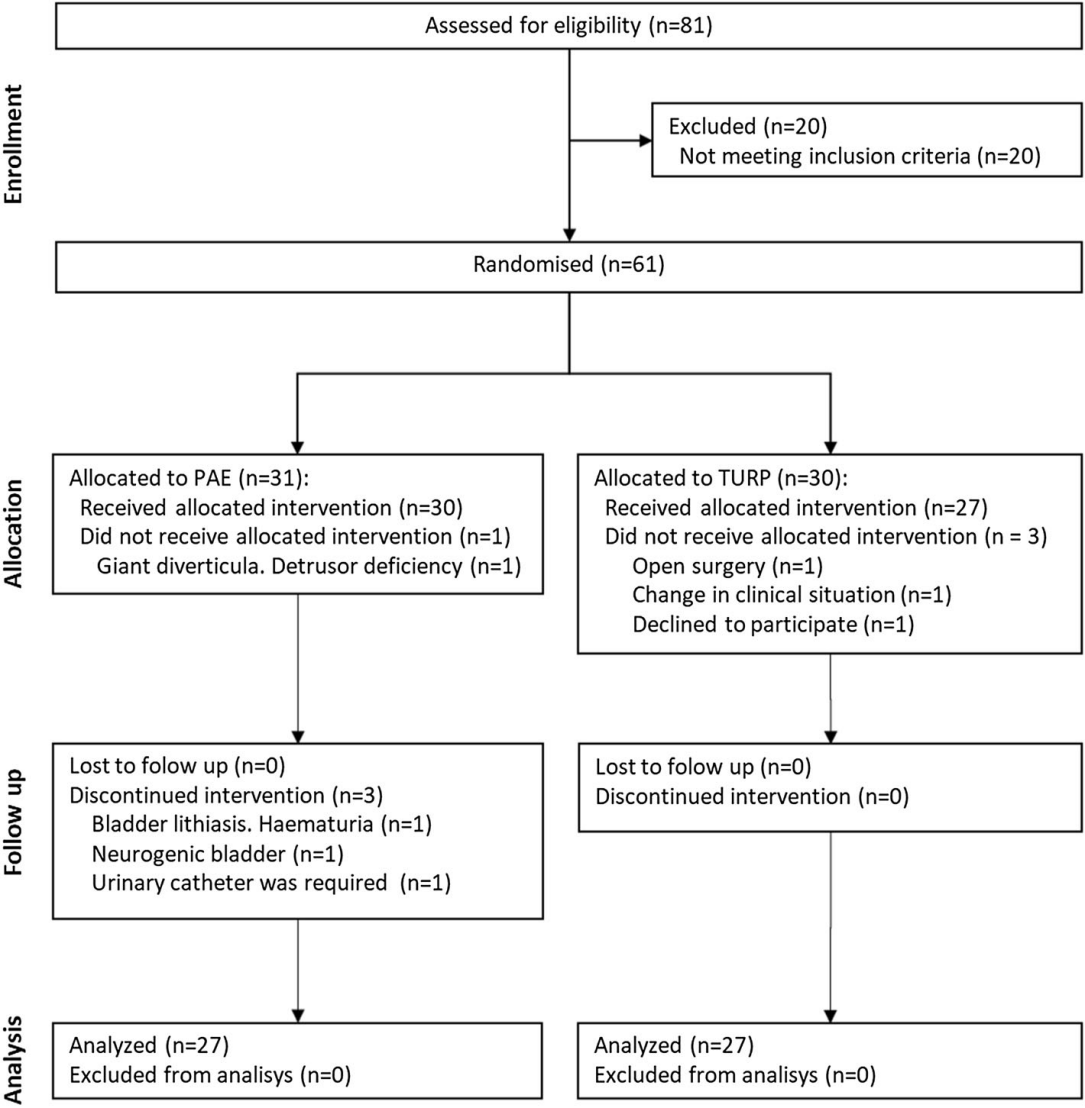
Patient flow chart

The data were obtained from an unblinded, single-center, non-inferiority, randomized, controlled trial performed in the Urology and Vascular and Interventional Radiology departments of the study site [3]. The trial was approved by the local Ethics Committee (code 49/2011), performed in accordance with the provisions of the Declaration of Helsinki [13] and the standards of Good Clinical Practice [14], and was registered in ClinicalTrials.gov (NCT01963312). Main inclusion criteria were men older than 60 years and indication for TURP. Eighty-one patients with LUTS secondary to BPH were assessed for study eligibility, and 20 patients did not meet the selection criteria detailed in the study protocol [15]. The patient flow chart is represented in Fig. 1.

The costs of each participant were categorized according to study the period: pre-intervention (from the informed consent to the operation), intervention (PAE or TURP), inpatient stay (from completion of the intervention to hospital discharge) and follow-up (from hospital discharge to the end of study). All costs were covered by Spanish National Health System and analyzed from a health care provider’s perspective.

During the pre-intervention and follow-up periods, patients received urology and interventional clinic (as specialized clinic), primary care clinic, as well as emergency care, when required. A computed tomography (CT) angiography was performed on all patients during the pre-intervention period as part of the trial’s selection criteria. The intervention costs were those associated with the procedure, histopathology and the stay in the recovery room. The costs of the PAE procedure item included: the professional fees of the interventional radiologist, the costs of the operation facilities (nursing services, technical staff, equipment, and imaging studies), and the medical supplies required for PAE (local anesthesia, catheters, microcatheters, guidewires, and microspheres). The costs of the TURP procedure item included: the professional fees of the urological surgeons, the costs of the operation facilities (nursing services and technical staff), the medical supplies required for TURP, and the costs of the anesthesia (anesthesiology staff, anesthetics drugs, and medical supplies). The costs of the inpatient stay included the physician’s professional fees, the nursing service fees, the medical supplies, the hospital pharmacy costs, the laboratory costs. Following hospital discharge, the patients from both groups were followed through scheduled visits at 1, 3, 6, and 12 months. Complications were graded using the Clavien classification system [16] and their associated costs as well as the outpatient prescriptions for BPH were also considered.

Staffing services costs; imaging studies, hospital room stays, the medical supplies, the hospital pharmacy costs, the laboratory costs and clinic visits were provided by the hospital’s Accounting Department. Administrative and accommodation (catering, laundry, and cleaning services) expenses were also included. The calculations were performed based on mean personal costs per hour, charges for room costs per hour, and the proportionate depreciation of equipment.

The resulting costs were summarized using means and standard deviations for continuous variables and frequencies and percentages for categorical variables. Differences between PAE and TURP in terms of their baseline characteristics, preoperative data, postoperative data, and costs were compared using Student’s *T* test and with the mid-*p* exact test in the case of complications. All comparisons were two-sided and a significance level of 0.05 was considered. Analyses were performed using software SPSS® Statistics version 22.0.

## Results

**Fig. 2:**
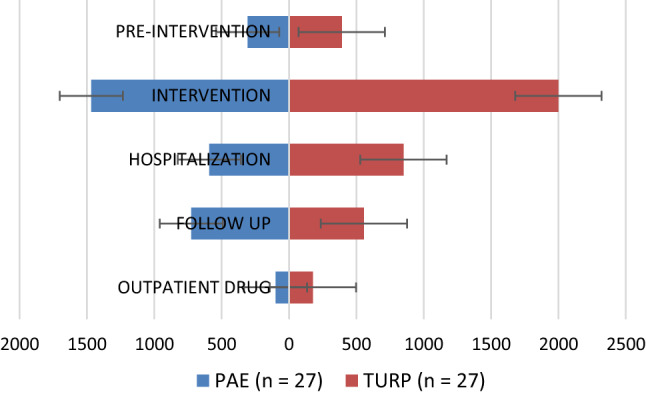
Summary of the mean patient costs for PAE and TURP. Abbreviations: PAE = prostatic artery embolization; TURP = transurethral resection of the prostate

A total of 61 patients were randomized in the trial: 31 to PAE group and 30 to TURP group. After patient exclusion due to not receiving the allocated intervention or discontinuing it, a final total of 54 patients completed the trial protocol and were included in the cost analysis: 27 of PAE group and 27 of TURP group (bipolar resection). The baseline characteristics of the study patients, as well as the preoperative and postoperative data, are described in Table 1. The mean total costs per patient were lower for PAE (€ 3,192.87) than TURP (€ 3,974.57), with this difference of € 781.70 being significant *(p* = 0.026). Detailed itemized costs arising from PAE and TURP are shown in Table 2 and summarized in Fig. 2.

**Table 1:** Baseline characteristics, perioperative, and postoperative data of the study patients

	PAE (*n* = 27)	TURP (*n* = 27)	*p*-value
*Baseline characteristics, mean(SD)*			
Age, years	72.11 (6.86)	72.26 (5.41)	0.925
Prostate volume, ml	75.27 (43.17)	77.91 (42.10)	0.847
PSA, ng/mL	4.32 (3.62)	5.87 (10.35)	0.484
Qmax, mL/s	7.23 (2.27)	7.13 (2.33)	0.900
IPSS, score	25.56 (5.18)	26.10 (6.23)	0.778
Length of follow-up, days	49.00 (58.90)	67.33 (32.96)	0.018
*Peri operative data*			
Anesthesia, *n* (%)			
General		1 (3.70)	
Spinal		26 (96.30)	
Local	27 (100.0)		
Procedure time, min; mean (SD)	143.70 (50.95)	74.81 (21.50)	< 0.001
Length of hospital stay, days; mean(SD)	1.00 (0.00)	1.67 (1.07)	0.003
Complications during the hospitalization, *n* (%)			
Clavien grade II	0 (0)	2 (100)	0.25
*Postoperative data*			
Mean length of the follow *n* (%)	376.33 (22.76)	360.00 (23.04)	0.003
Complications during the follow-up	17	47	< 0.0011
Clavien grade I	5 (29.41)	27 (57.45)	
Clavien grade II	12 (70.59)	19 (40.43)	
Clavien grade III		1 (2.03)	

^1^mid-*p* exact test

Abbreviations: PAE = prostatic artery embolization; TURP = transurethral resection of the prostate; SD = standard deviation

**Table 2:** Cost breakdown arising from PAE and TURP

	PAE (*n* = 27)		TURP (*n* = 27)		*p*-value
	Mean (€)	SD	Mean (€)	SD	
Pre-intervention(total)	307.37	130.17	391.47	85.53	0.015
Specialized clinic^a^	66.59	34.54	84.25	31.73	0.063
Primary care clinic	20.96	33.26	18.51	28.01	0.752
CT angiography	148.41	109.97	127.85^b^	47.18	0.361
Transabdominal ultrasonography	29.63	9.91	28.33	10.53	0.649
Laboratory	19.96	13.66	38.96	12.13	< 0.001
X-ray^c^	0.00	0.00	14.33	8.20	< 0.001
Pre-anesthesia consultation^c^	0.00	0.00	72.53	1.28	< 0.001
Urologic emergency^d^	21.81	62.88	6.70	34.83	0.298
Intervention (total)	1468.00	319.21	1999.70	489.94	< 0.001
Procedure	1468.00	319.21	1684.25	464.06	0.061
Laboratory (histopathology)	0.00	0.00	152.22	27.82	< 0.001
Recovery room	0.00	0.00	163.23	107.24	< 0.001
Inpatient stay (total)	591.79	95.45	849.43	439.20	0.004
Inpatient room	576.37	95.014	832.50	489.94	0.003
Laboratory	4.67	8.02	0.81	4.23	0.043
Hospital pharmacy	10.33	5.31	7.98	5.97	0.022
Complications	0.00	0.00	7.81	31.09	0.222
Total in-hospital costs	2059.79	379.86	2849.13	826.30	< 0.001
Follow-up (total)	725.26	1546.57	556.22	522.60	0.605
Specialized clinic	231.33	65.99	197.79	61.78	0.065
Primary care clinic	56.41	54.58	78.56	67.78	0.212
Laboratory	77.52	36.40	85.30	52.88	0.552
Complications	360.00	1482.36	194.58	398.65	0.586
Outpatient drug (total)	100.45	80.15	177.75	77.17	< 0.001
Total costs	3192.87	1564.96	3974.57	930.68	0.026

^a^ Specialized clinic: urology and/or intervention radiologist clinic

^b^ Mandatory for clinical trial selection criteria. In the context of standard of care should not be considered for TURP

^c^ Preoperative evaluation only for TURP according to the routine practice

^d^ Urologic emergency: any urological condition that require urgent assistance

Significant cost differences between PAE and TURP were observed during the pre-intervention period (€ 307.37 and € 391.47, respectively, *p* = 0.015). The differences were observed for the pre-anesthesia items required prior to TURP procedure. No significant differences were observed in the mean costs of PAE (€ 1,468.00) and TURP (€ 1,684.25) procedures (*p* = 0.061). However, the recovery room stay and the histopathology substantially increased the total interventional itemized costs for TURP (€ 1,999.70) compared with PAE (€ 1,468.00) (*p* < 0.001). Significant differences in costs were also observed during the post-intervention hospital stay: € 591.79 for PAE and € 849.43 for TURP (*p* = 0.004). The mean total in-hospital costs, that included intervention and inpatient stay, was found to be significantly greater for TURP population compared with PAE population (€ 2,849.13 vs € 2,059.79; *p* < 0.001).

The mean follow-up was 376.33 days for PAE and 360.00 days for TURP (*p* = 0.003) and no significant differences were observed in the mean costs. During this period, complications appeared in both groups (17 for PAE and 47 for TURP) and accounted for 49.64% of the total follow-up cost in PAE population and for 34.98% in TURP population. The differences between both groups in this respect were not significant. The outpatient prescriptions for the urological process consisted of *α*1-blockers, 5*α*-reductase inhibitors, urinary antispasmodics, analgesics, antibiotics, low-molecular-weight heparin (LMWH), and incontinence pads or urine collection bags. The costs of the outpatient prescriptions were significantly higher in TURP group (€ 177.75 vs € 100.45; *p* < 0.001).

## Discussion

Available evidence suggests that PAE is an effective and safe technique for the treatment of LUTS secondary to BPH, with clinical outcomes comparable to those of TURP. However, TURP seems to be superior when considering urodynamic parameters such as Qmax and post-void residual (PVR) [3, 4, 17]. In this study, we evaluated the costs of PAE and TURP in a randomized controlled clinical trial. The analysis was performed from the perspective of a hospital of the Spanish National Health System and included resources consumed by each patient in relation to their urological process (from primary and specialized care settings) during the period elapsed from the date of signature of the informed consent to completion of the follow-up and the end of study visit. The analysis estimated that the total costs of PAE were 19.67% lower than those of TURP, with the difference being statistically significant.

To date, two comparative in-hospital cost analyses between PAE and TURP have been published: Bagla et al. [11] carried out a retrospective review comparing hospital costs, and Müllhaupt et al. [12] performed a cost analysis based on data obtained from an unblinded, single-center, randomized, controlled study. Both studies took into account the variables and costs related to the intervention and inpatient stay (hospitalization), but not the items involved during the pre-intervention period and follow-up. These authors reported lower in-hospital costs for PAE group and a shorter hospitalization, both of which are consistent with the results of our study. In line with the findings of Bagla et al., but in contrast with the results of Müllhaupt et al., our data indicated that the total costs associated to the interventions (surgical procedure, recovery room stay, and histopathology analysis) were also significantly lower in PAE group, specifically 26.59% lower, compared with TURP group. The lower cost of PAE was due that was performed under local anesthesia and, without the need for an operating room, a recovery room, and anesthesiology staff.

Inpatient stay costs were also significantly higher for TURP. The excess costs could be related to longer hospital stay compared with PAE (1.67 vs 1 day, including an overnight stay; *p* = 0.003). Nevertheless, the mean length of hospitalization for TURP observed in our study is shorter than that published in the literature [18].

According to the clinical protocol, the patients were followed-up through scheduled visits over a period of 12 months in specialized care and primary care clinics. During this period, complications related to the urological process appeared in both groups, including hematuria, urinary tract infection, urinary retention, rectal ischemia, and urethral stricture. These complications substantially contributed to the follow-up costs in both groups. PAE cost resulting from complications was mainly associated with a patient who developed transient rectal ischemia after the intervention. This patient was managed with conservative treatment, had to visit the emergency care clinic on multiple occasions and required a prolonged hospital stay. Despite this, the differences in the follow-up period costs were not statistically significant.

Outpatient prescriptions were another cost factor that was considered during the study period. All patients of TURP group received thromboprophylaxis with LMWH for ten days after the surgery according to the standard of care protocol in force in the trial site. This drug accounted for 30.85% of the cost of the outpatient prescriptions for patients of TURP group and was consequently the main cause of the significant excess cost calculated for this procedure compared with PAE.

It is essential to bear in mind that this study has several limitations, including the fact that it was performed as a post hoc analysis, which has inherent limitations. In addition, the data were obtained from a randomized clinical trial carried out with patients who were protocol-linked and operated on in a single site. To accurately reflect routine clinical practice, costs of CT angiography of TURP group and PAE inpatient room should not be considered. PAE is currently performed as outpatient settings, which results in considerably lower costs by removing the need for hospitalization. Considering these conditions, significant differences in mean total costs per patient were still significantly lower for PAE (€ 2,904.68 vs € 3,846.72; *p* = 0,009). Another limitation of the study is that thromboprophylaxis with LMWH is site routine practice but is not endorsed by the European Association of Urology (EAU) guidelines, so may differ from that applied in other healthcare frameworks. Although the costs related to the depreciation of equipment, electricity, security, medical records, and housekeeping were included, those of lost productivity, unpaid work (domestic), or lost leisure time were not. Some studies suggest that re-intervention rates in PAE are clearly higher than in TURP due to persistent LUTS. According to the data of the UK Registry of Prostate Embolization (UK-ROPE) study [7], PAE was associated with a re-intervention rate of 19.9% within 2 years, whereas only 5% of men who had undergone an initial TURP procedure needed repeat surgery. Although this issue could substantially increase the costs of PAE, none of our study patients required a repeat intervention during the follow-up period.

## Conclusions

For most items, the analyzed costs related to TURP were significantly higher than those of PAE in the context of this clinical trial with a short-term follow-up. The most relevant costs for both procedures were those arising during the interventional period, with significant differences being found between them. However, no significant differences were observed during the 12-month follow-up, including in terms of the secondary complications. Future investigations in the context of routine clinical practice should be aimed at comparing the long-term effectiveness of both procedures and determining their cost-effectiveness.
